# Assessment of Cardiac Toxicity of Manganese Chloride for Cardiovascular Magnetic Resonance

**DOI:** 10.3389/fphys.2022.952043

**Published:** 2022-07-07

**Authors:** Elodie Lamonzie, Fanny Vaillant, Emma Abell, Sabine Charron, Dounia El Hamrani, Bruno Quesson, Fabien Brette

**Affiliations:** ^1^ Univ, Bordeaux, CRCTB, Inserm, Bordeaux, France; ^2^ IHU Liryc, Electrophysiology and Heart Modeling Institute, Bordeaux, France

**Keywords:** MRI, manganese, cardiac function, electrophysiology, toxicity, calcium

## Abstract

MRI is widely used in cardiology to characterize the structure and function of the heart. Currently, gadolinium-based contrast agents are widely used to improve sensitivity and specificity of diagnostic images. Recently, Manganese, a calcium analogue, has emerged as a complementary contrast agent with the potential to reveal remaining viable cells within altered tissue. Imaging applications may be limited by substantial toxicity of manganese. Indeed, cardiac safety of manganese is not yet comprehensively assessed. In this study we investigated the effect of MnCl_2_ (1–100 µM) on cardiac function. Hemodynamic function was determined *ex vivo* using an isolated working rat heart preparation. HL-1 cardiac myocytes were used to investigate cell viability (calcein AM) and calcium cycling (Cal-520 a.m.). Rat ventricular cardiomyocytes were dissociated by enzymatic digestion. Action potentials and calcium currents were recorded using the patch clamp technique. MRI experiments were performed at 1.5T on formalin-fixed rat hearts, previously perfused with MnCl_2_. MnCl_2_ perfusion from 1 up to 100 µM in isolated working hearts did not alter left ventricular hemodynamic parameters. Contractility and relaxation index were not altered up to 50 µM MnCl_2_. In HL-1 cardiac myocytes, incubation with increasing concentrations of MnCl_2_ did not impact cell viability. The amplitude of the calcium transients were significantly reduced at 50 and 100 µM MnCl_2_. In freshly isolated ventricular myocytes, action potential duration at 20, 50 and 90% of repolarization were not modified up to 10 µM of MnCl_2_. L-type calcium current amplitude was significantly decreased by 50 and 100 µM of MnCl_2_. MRI on heart perfused with 25 and 100 µM of MnCl_2_ showed a dose dependent decrease in the T1 relaxation time. In conclusion, our results show that low concentrations of MnCl_2_ (up to 25 µM) can be used as a contrast agent in MRI, without significant impact on cardiac hemodynamic or electrophysiology parameters.

## Introduction

Cardiac MRI is a non-invasive clinical imaging technique allowing characterization of various heart diseases. Currently, gadolinium-based contrast agents are the gold standard since they allow increasing contrast between healthy and altered myocardium using specific acquisition method called Late Gadolinium Enhancement MRI (LGE-MRI). Manganese has atomic structural similarities to calcium and can enter cardiomyocytes through voltage-gated calcium channels, allowing intracellular distinction of living cells (Ochi, 1976). Manganese-based contrast agents may thus provide different information than LGE-MRI for various cardiac pathologies, allowing for example visualization of remaining viable tissues inside or around infarcted regions (Hu et al., 2005; Bianchi et al., 2017; Tada et al., 2019). However, high concentration of manganese increases the competition between Mn^2+^ and Ca^2+^ ions on calcium channels which can lead to myocardial depression (for review see (Brurok et al., 1997)). Since the 70s, numerous studies have investigated the impact of MnCl_2_ on cardiac excitation-contraction coupling (Delahayes, 1975) and action potentials (Takeya and Reiter, 1972; Ochi, 1975). Biophysical studies showed that manganese decreases the amplitude of calcium currents was observed in Guinea Pigs (Ochi, 1976). However a fully comprehensive study of the cardiac toxicity of manganese is missing. Indeed, manganese neurotoxicity has been observed at high manganese exposure, causing a neurological syndrome similar to Parkinson’s disease called manganism such as cognitive abnormalities and motors disorders, principally bradykinesia, hypertonia and tremors (Cersosimo and Koller, 2006). A manganese accumulation in the brain is observed mainly in the basal ganglia, specifically in globus pallidus (Criswell et al., 2012) but also in the frontal cortex (Ma et al., 2018). Despite these studies, the European Commission authorized the marketing of Teslascan (Mangafodipir, Mn-DPDP), a chelated manganese that reduces manganese ion toxicity, as a contrast agent for the detection of hepatic metastatic lesions (Bartolozzi et al., 2004) or hepatocellular carcinoma (Murakami et al., 1996) but also pancreatic lesions (Wang, 1998), with a dosage of 5 μmol/kg (Federle et al., 2000).

Recently, cardiac MRI using manganese have started in animals but also in humans in clinical trials (Daire et al., 2008; Dash et al., 2011; Tada et al., 2019; Jasmin et al., 2021; Spath et al., 2021). It is therefore crucial to investigate the possible cardiac toxicity of manganese. Here, we carefully investigated the effect on MnCl_2_ on cardiac function at the whole heart level but also at cellular level. Finally, we evaluated if a safe concentration of MnCl_2_ could be used in cardiac MRI.

## Material and Methods

### Ethics

All experiments were conducted in accordance with the European Union directive EU/2010/63. Local ethical approval was obtained from the Ethical committee of Bordeaux, France.

### Rat Heart Perfusion in the Semi Recirculating Working Mode

Male Wistar rats (289 ± 5 g, Janvier Laboratory, France) were housed in controlled environment (12/12 h light/dark, temperature 21–23°C, humidity 40/60%), with food and water *ad libitum*. Rats were anesthetized with isoflurane (3% for 5 min) and were heparinized (5000 UI/Kg s. c. subcutaneous) 5 min before their euthanasia by cervical dislocation. The heart was rapidly removed and perfused *ex vivo* in the working mode with modified Krebs-Henseleit solution (containing (in mmol/L): 116 NaCl, 5 KCl, 1.2 MgSO_4_, 1.2 NaH_2_PO_4_, 27 NaHCO_3_, 5.5 glucose, 1.8 CaCl_2_, 0.2 pyruvate, 1 lactate, 0.008 insulin) gassed with 5% CO_2_ and 95% O_2_ (pH 7.4) at 37.5°C. The preload and afterload were maintained at 11 and 70 mmHg, respectively. The cardiac functions (left ventricular hemodynamic, cardiac flows, heart rate) were continuously recorded throughout the experiment, by an EMKA-IOX2 data acquisition system (EMKA Technologies, Paris, France).

**FIGURE 1:**
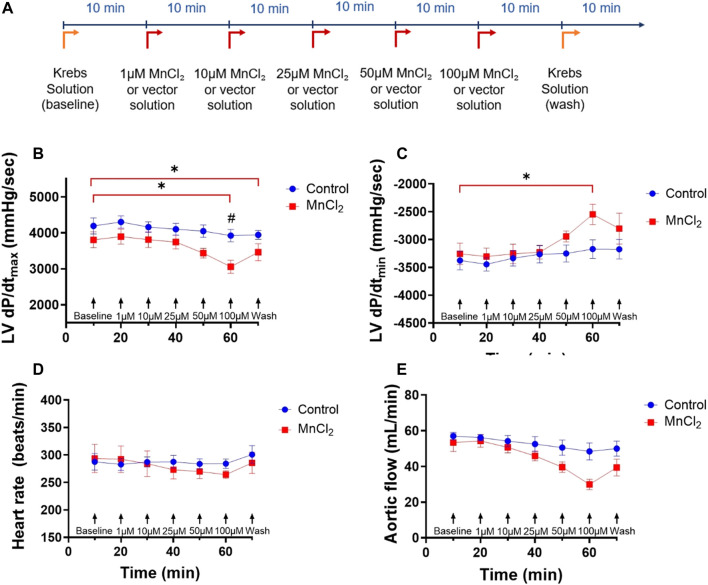
Effects of MnCl_2_ on left ventricle hemodynamic properties of rat hearts perfused in working mode. **(A)** Perfusion protocol for working hearts with or without MnCl_2_ **(B)** dP/dt_max_ **(C)** dP/dt_min_ of left ventricle **(D)** heart rate **(E)** aortic flow depending on time for the control group (n = 5) (blue dots) and the group MnCl_2_ (*n* = 6) (red dots). *p < 0.05 vs. baseline of the same group. ^#^ *p* < 0.05 vs. same condition of control group.

Two groups were carried out: a control group (N = 5) and a group perfused with MnCl_2_ (N = 6) for 70 min. In MnCl_2_ group, hearts were perfused with a Krebs-Henseleit buffer without MnCl_2_ for 10 min (baseline), and with increasing concentrations of MnCl_2_ (1, 10, 25, 50 and 100 µM) added every 10 min. During the last 10 min, all hearts were perfused with a Krebs-Henseleit buffer without MnCl_2_ to determine the reversibility of effects of MnCl_2_ on cardiac function. Controlled hearts underwent a perfusion time equal to MnCl_2_-treated hearts, with an equivalent volume of MnCl_2_ vehicle added every 10 min (Figure 1A). The impacts of MnCl_2_ on aortic, cardiac and coronary flow rates, left ventricular function and heart rate were assessed.

### Cell Viability

HL-1 cardiac cells were incubated with calcein-AM (2 µM) in physiological saline solution (in mmol/L: 140 NaCl, 5 KCl, 20 HEPES, 10 glucose, 1 MgCl_2_, 1 CaCl_2_, pH 7.4 with NaOH) for 30 min at 37°C in the dark. Dishes containing HL-1 cells were placed in an inverted microscope (Olympus, Japan; ×10 objective). Fluorescence signals were recorded after excitation at 488 nm and emission at >525 nm. A perfusion system allowed us to change solution from physiological saline solution to MnCl_2_ (1, 10, 25, 50 and 100 µM sequentially at 5 min interval) at room temperature (20–22°C). For each condition, two different regions of interest (ROI) were analyzed to measure fluorescence with Metamorph software (Molecular Devices, San Jose, United States).

### Calcium Imaging

HL-1 cardiac cells were loaded with a calcium sensitive dye (Cal-520 a.m., 10 µM) in physiological saline solution (in mmol/L: 140 NaCl, 5 KCl, 20 HEPES, 10 glucose, 1 MgCl_2_, 1 CaCl_2_, pH 7.4 with NaOH) for 30 min at 37°C in the dark. Cal-520 stock solution was prepared daily at 1 mM in 20% pluronic acid plus DMSO. Glass dishes containing HL-1 were placed in an inverted microscope (Olympus, Japan) at the ×10 objective. Cal-520 a.m. was excited at 488 nm and emission was collected at >525 nm. A series of images (∼30 frame per second, confocal microscope spinning disk; Yokogawa W-1) was recorded using Metamorph software. A perfusion system allowed us to change solution from physiological saline solution to MnCl_2_ (1, 10, 25, 50 and 100 µM sequentially at 5 min interval) at room temperature (20–22°C). Five regions of interest (ROI) were defined for each condition (one HL-1 cell/ROI). Frequency, calcium transient amplitude (F/F_0_) and the temporal evolution of calcium transients (time to peak and time to 50% decay) were measured using LabChart software.

### Rat Ventricular Myocytes Isolation

Male Wistar rats (299 ± 13 g, Janvier Laboratory, France) (N = 7) were euthanized as described above. Hearts were rapidly removed and perfused retrogradely with an isolation solution containing (in mmol/L): 120 NaCl, 5.4 KCl, 1.2 NaH_2_PO_4_, 10 HEPES, 10 glucose, 1.2 MgCl_2_, 30 taurine, 10 creatine, pH 7.3 with NaOH, supplemented with 0.75 CaCl_2_, at 37°C. After the coronary circulation had cleared of blood and the recovery of spontaneous and regular contractions, perfusion was continued with Ca-free isolation solution (isolation solution with 0.1 mM de EGTA) for 4 min, followed by enzymatic digestion (isolation solution containing 50 µM CaCl_2_, 0.8 mg/ml collagenase type II and 0.08 mg/ml protease type XIV) for 15 min. The ventricles were then excised from the heart, minced, and gently shaken at 37°C in collagenase-containing solution supplemented with 1% bovine serum albumin. Ventricular cells were filtered from this solution at 5-min intervals and resuspended in isolation solution containing 1 mM Ca^2+^.

### Electrophysiological Recording

Isolated myocytes were studied in a chamber mounted on the stage of an inverted microscope (Olympus, Japan) and cells were perfused with control physiological solution containing (in mmol/L): 140 NaCl, 5 KCl, 20 HEPES, 10 glucose, 1 MgCl_2_, 1 CaCl_2_, pH 7.4 with NaOH. All experiments were performed at room temperature. Electrophysiological data was recorded using a Digidata 1,550 controlled by an Axopatch 200B amplifier running pClamp software (Axon Instruments, Molecular Devices). Patch pipette resistance was 2.5–3.5 MΩ. Action potentials (APs) were evoked using 2 ms supra-threshold current steps at a frequency of 0.5 Hz. The pipette solution contained (in mmol/L): 130 K-glutamate, 9 KCl, 10 NaCl, 0.5 MgCl_2_, 5 Mg-ATP, 0.5 EGTA, 10 HEPES, 0.4 GTPTris, set to pH 7.2 with KOH. The bath solution was the normal physiological salt solution, followed by different concentrations of MnCl_2_ (1, 10, 25, 50 and 100 µM).

I_CaL_ was elicited using a voltage step of 500 ms to 0 mV from holding potential of −80 mV. To specifically measure I_CaL_, the external solution containing (in mmol/L): 130 TEA, 0.5 MgCl_2_, 1 CaCl_2_, 10 HEPES, 10 glucose, pH 7.4 with TEA-OH. The pipette solution containing (in mmol/L): 120 CsCl, 20 TEA-Cl, 1 MgCl_2_, 5 Mg-ATP, 0.4 GTP-Tris, 10 HEPES, 5 EGTA, 0.1 CaCl_2_, pH 7.2 with CsOH. These Na^+^- and K^+^-free external and internal solutions allow us to avoid contamination by overlapping ionic currents, and to use a physiological holding potential (Brette et al., 2006). Trains of depolarizing pulses were applied at 0.1 Hz. A fast perfusion system placed close to the cell was used to deliver the external solution.

AP amplitude was measured as the difference between the overshoot and the resting membrane potential. The maximum rate of rise of the AP (dV/dtmax) was calculated by differentiation of the AP upstroke using Clampfit software. Action potential duration (APD) was measured as the duration from the overshoot to 20, 50 and 90% of repolarization (APD_20_, APD_50_ and APD_90_, respectively). I_CaL_ amplitude was measured as the difference between peak and the end of depolarizing pulse current. I_CaL_ are expressed as current density by normalizing to cell size (pA/pF).

### MRI Acquisitions and Post-Processing

Wistar rat hearts were removed and placed in the perfusion system in working mode (described above). A 15 min cardiac perfusion was performed with Krebs saline solution or Krebs saline solution at 25 µM or 100 µM of MnCl_2_ (N = 4/group). Then, each heart was fixed in Langendorff system with formalin solution during 30 min. Formalin was removed from the heart cavities and replaced with Fluorinert (Electronic liquid FC-770, 3M Electronics, St. Paul, United States) for the MRI session. Fluorinert is a perfluoropolyether chemical, undetectable in MRI which allows to reduce air-tissue artefacts on MR images.

MR images were acquired at 1.5T system (MAGNETOM Aera, Siemens, Erlangen, Germany) with a custom made 2 cm diameter receive-only loop coil (designed using a 35 µm-thick copper trace on a FR4 substrate.

A 3D FLASH sequence was performed to obtain scout images and to determine the short axis view of the rat heart: TE/TR = 11/18 ms; flip angle = 15°; FOV = 80 × 50 mm; matrix = 176 × 110; resolution in plane = 0.5 × 0.5 mm^2^; 32 slices; slice thickness = 1 mm, bandwidth = 171 Hz/pixel.

T1 measurements were performed using successive inversion recovery spin echo sequences with one slice positioned at mid-ventricular short axis of the heart: TE/TR = 10/763 ms; flip angle = 90°; FOV = 95 × 95mm; matrix = 64 × 64; resolution = 1.5 × 1.5 × 1.5 mm^3^, bandwidth = 797 Hz/pixel. The inversion times (TI) were as followed = 25; 50; 75; 100; 150; 200; 250, 300, 400, 500 and 750 ms. T1 values were calculated using a custom software written in Matlab (Mathworks, ver. R2020a) and a region of interest (ROI) was drawn on the left ventricle.

### Chemicals

All solutions were prepared using ultrapure water supplied by a Milli-Q system (Millipore, United States).

All chemical products were bought from Sigma (St. Louis, MO) except for Cal-520 (AAT-Bioquest, Sunnyvale, United States) and collagenase type II (Worthington Biochemical, Lakewood, United States).

### Statistical Analysis

Data are reported as mean ± SEM. Statistical analysis was performed using GraphPad Prism eight software. Two-way ANOVA with Dunett post-hoc analysis was used after confirmation of a normal distribution. If data was not normally distributed, Friedman test was used for paired data and Kruskal–Wallis test was used for unpaired data. For T1 measurements, one-way ANOVA with *post-hoc* test of Tukey was performed. *p* < 0.05 was considered statistically significant.

## Results

### Effects of Manganese on Cardiac Functional Properties

We first determined the effect of MnCl_2_ on hemodynamic parameters during *ex vivo* perfusion of rat hearts. Figure 1 top panel shows the experimental protocol. Low micromolar concentration of MnCl_2_ (up to 50 µM) did not alter heart rate, LV dP/dt_max_, LV dP/dt_min_ or aortic flow (Figures 1B–E). We observed a small but significant effect of 100 µM MnCl_2_ on LV dP/dt_max_, LV dP/dt_min_ and aortic flow (Figures 1B,C,E). Other functional parameters such as maximum left ventricular systolic pressure, minimal left ventricular pressure, end diastolic pressure, left ventricular develop pressure were not modified by micromolar concentration of MnCl_2_ except for cardiac power and stroke volume at 100 µM and from 50 μM MnCl_2_, respectively (Table 1). Importantly, the slight effect of high micromolar concentration MnCl_2_ on perfused hearts was reversible and temporary (Figure 1; Table 1). Indeed, during reperfusion of hearts having received ascending concentrations of MnCl_2_ with a Krebs solution without MnCl_2_ (wash), hemodynamic parameters were similar to the control values (Figure 1; Table 1), except for a slight decrease in LV dP/dt_max_. Finally, we checked for possible gross structural alteration (*e.g.,* edema) by weighing the hearts at the end of the procedure. No significant difference was observed after the perfusion, on heart weight between control (1.39 ± 0.06 g) and MnCl_2_ group (1.39 ± 0.07 g). Taken together, these data indicate that low micromolar concentration of MnCl_2_ does not alter cardiac function on *ex-vivo* heart working mode.

**TABLE 1:** Hemodynamic parameters of isolated working rat hearts perfused in the control group and MnCl_2_ group**.**

	–	Baseline	1 µM	10 µM	25 µM	50 µM	100 µM	Wash
**LVSPmax (mmHg)**	**Control**	110 ± 3	113 ± 2	111 ± 2	109 ± 3	109 ± 3	107 ± 3	107 ± 2
	**MnCl**_**2**_	106 ± 3	109 ± 3	108 ± 3 *	108 ± 3	105 ± 3	99 ± 4	105 ± 5
**LVPmin (mmHg)**	**Control**	3.67 ± 0.49	3.45 ± 0.40	3.56 ± 0.49	3.72 ± 0.51	3.72 ± 0.53	3.89 ± 0.57	3.94 ± 0.59
	**MnCl**_**2**_	4.67 ± 0.92	4.39 ± 0.76	4.89 ± 0.86	5.28 ± 0.72	5.22 ± 0.76	6.11 ± 0.48	5 ± 0.89
**LVeDP (mmHg)**	**Control**	7.78 ± 0.8	7.83 ± 0.72	9.28 ± 0.64	10 ± 1.06	9.56 ± 0.95	10.17 ± 0.99	9.83 ± 1.40
	**MnCl**_**2**_	7.95 ± 0.85	7.83 ± 0.72	9.28 ± 0.64	10 ± 1.06	9.56 ± 0.95	10.17 ± 0.99	9.22 ± 1.5
**LVDP (mmHg)**	**Control**	107 ± 3	110 ± 2	107 ± 2	106 ± 3	106 ± 3	103 ± 3	103 ± 2
	**MnCl**_**2**_	101 ± 3	104 ± 3	103 ± 3	103 ± 3	100 ± 3	93 ± 4	99 ± 5
**Cardiac Output (ml/min)**	**Control**	76.14 ± 5.65	75.76 ± 4.09	73.15 ± 8.19	71.33 ± 10.43	68.99 ± 11.4	66.64 ± 13.05	67.34 ± 11.15
	**MnCl**_**2**_	73.3 ± 13.53	74.8 ± 10.91	70.66 ± 10.55	64.76 ± 10.51	57.78 ± 12.11	47.94 ± 10.86	58.4 ± 16.67
**Coronary flow (ml/min)**	**Control**	19.12 ± 1.38	19.22 ± 1.37	18.97 ± 1.24	18.81 ± 1.08	18.46 ± 1.06	18.21 ± 1.17	17.24 ± 1.58
	**MnCl**_**2**_	19.93 ± 2.17	20.51 ± 2.18	20.00 ± 2.38	18.92 ± 2.36	18.19 ± 2.55	17.99 ± 2.88	19.15 ± 2.40
**SV (mL/beat)**	**Control**	0.267 ± 0.011	0.268 ± 0.012	0.256 ± 0.014	0.25 ± 0.018	0.245 ± 0.019	0.236 ± 0.02	0.225 ± 0.016
	**MnCl**_**2**_	0.255 ± 0.019	0.262 ± 0.018	0.251 ± 0.024	0.237 ± 0.024	0.217 ± 0.027	0.176 ± 0.022 **	0.208 ± 0.035
**Cardiac power (mWatts)**	**Control**	17.99 ± 0.39	18.39 ± 0.50	17.49 ± 1.07	16.84 ± 1.41	16.26 ± 1.45	15.47 ± 1.61	15.79 ± 1.37
	**MnCl**_**2**_	16.50 ± 1.37	17.23 ± 0.95	16.17 ± 1.07	14.72 ± 0.96	12.71 ± 1.06 *	9.92 ± 0.99 *	12.96 ± 1.76

*p < 0.05, **p < 0.01 vs. baseline of the same group. LVSP_max_, maximum left ventricular systolic pressure; LVP_min_, minimal left ventricular pressure; LVeDP, left ventricular end diastolic pressure; LVDP, left ventricular developed pressure; HR, heart rate; SV, stroke volume.

Bold Values as described in the protocol (Figure 1A).

### Manganese Does Not Affect Cell Viability

**FIGURE 2:**
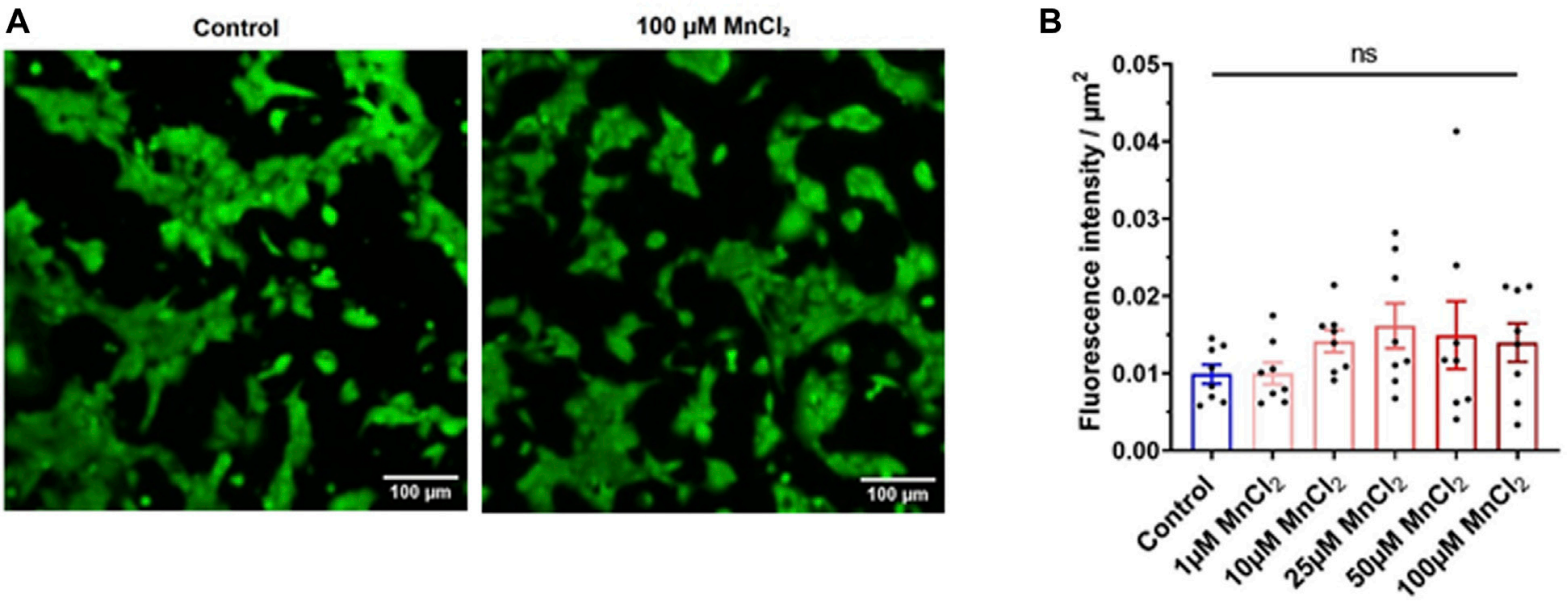
Effect of MnCl_2_ on the cell viability of HL-1 cells. **(A)** Representative image of the calcein fluorescence in HL-1 cells in control (left) and 100 µM MnCl_2_ (right) condition. **(B)** Total fluorescence of calcein expressed par µm^2^ between the control condition and the different MnCl_2_ concentrations (*n* = 8). ns, no significant.

We next tested the possibility that manganese toxicity induces cell death. Calcein-AM loaded HL-1 cardiac myocytes were used to determine cell viability. Calcein is retained in the cell with intact membranes, whereas it is released from dead cells (Neri et al., 2001). Therefore, cell fluorescence is an indicator of the cell viability. Figure 2A shows representative images of HL-1 under control condition and bathed with 100 µM MnCl_2_. Mean data indicates that micromolar concentration of MnCl_2_ did not affect viability of HL-1 cardiac myocytes.

### Effect of Manganese on Calcium Transients

**FIGURE 3:**
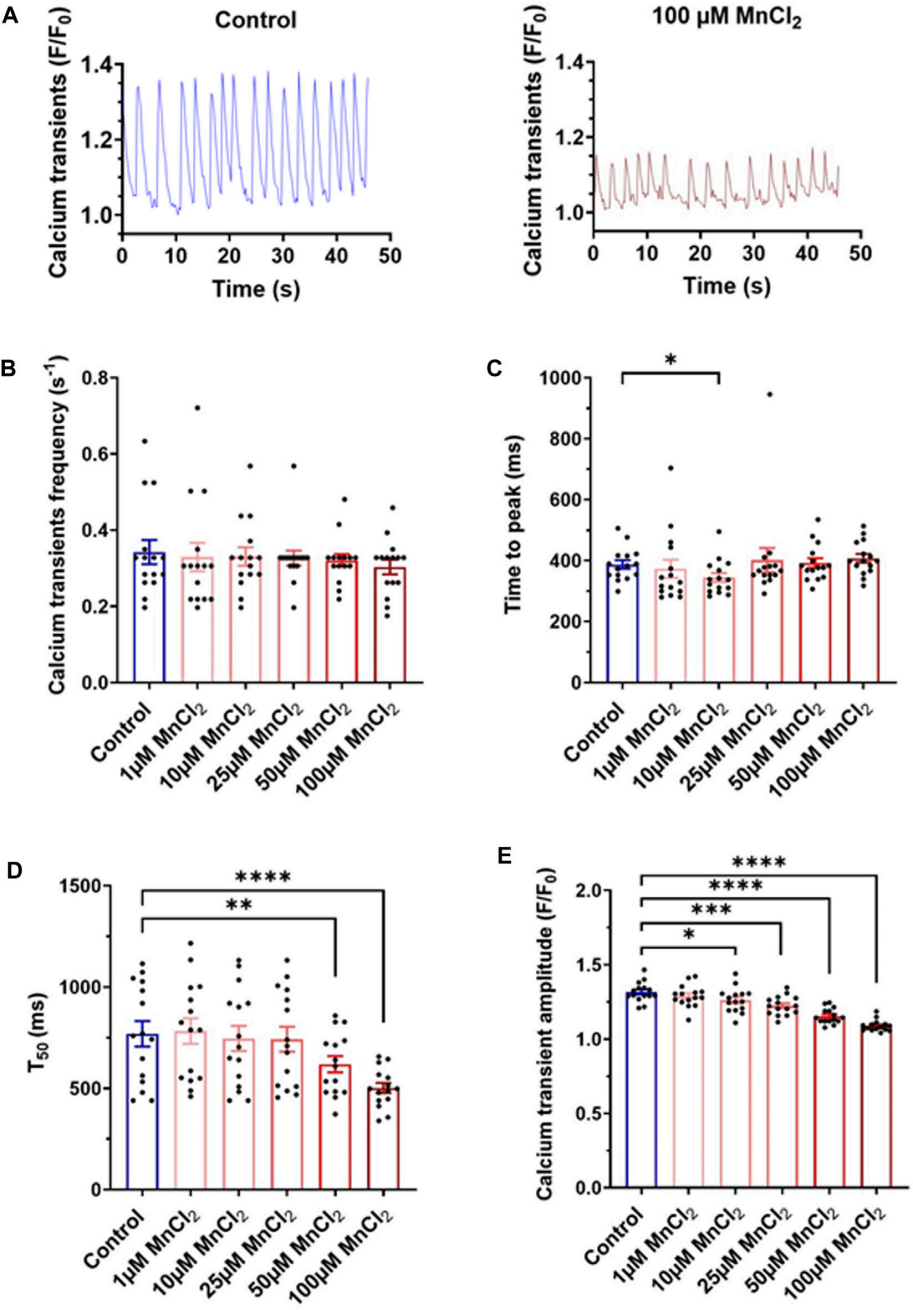
Variation of calcium transients induced by MnCl_2_ in HL-1 **(A)** Representative plots of calcium transients in HL-1 observed on control solution (left) and at 100 µM of MnCl_2_ (right). **(B)** Frequency **(C)** time of peak **(D)** time at 50% of the relaxation (T_50_) and **(E)** amplitude of calcium transients depending on different conditions (*N* = 3, *n* = 15). *p < 0.05, **p < 0.01.

Albeit micromolar concentration of MnCl_2_ does induce cell death, we next investigated a possible acute toxicity at the cellular level on physiological function. We first recorded Ca^2+^ transient using confocal microscopy. HL-1 cardiac cells display calcium oscillations under basal condition (Figure 3A). Frequency and the time to peak of these Ca^2+^ transient were not modified by MnCl_2_ (Figures 3B,C), whereas time to 50% decay was significantly reduced from 50 µM MnCl_2_ (Figure 3D). A dose dependent decrease in calcium transient amplitude was observed (Figure 3E). Albeit statistically significant, amplitude reduction was low with 1, 10 and 25 µM MnCl_2_ (2.1 ± 0.6%, 4.2 ± 0.7% and 7.0 ± 0.6%, respectively). This reduction was further marked at 50 and 100 μM MnCl_2_, reaching 12.2 ± 0.9% and 17.1 ± 1.0%, respectively. Such a reduction can be explained by fluorescence quenching with MnCl_2_, but also by blocking of L-type Ca^2+^ channel by MnCl_2_, crucial in determining the size of cytosolic Ca^2+^ transient. Therefore we next investigated the effect of high micromolar MnCl_2_ on L-type Ca^2+^ current.

### High Micromolar MnCl_2_ Decreases Calcium Current Amplitude

**FIGURE 4:**
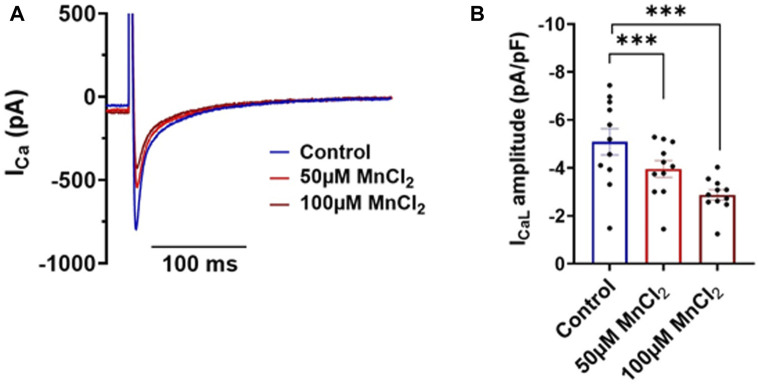
Effects of MnCl_2_ on calcium currents of rat ventricular cardiomyocytes **(A)** Representative plots of calcium currents in control condition (blue line) and at 50 µM (light red line) and at 100 µM (dark red line) of MnCl_2_ (the capacitance of the cell is 107 pF) **(B)** Calcium current density in depending on different conditions. *N* = 3, *n* = 11. ***p < 0.001.

Using a more translational approach, we recorded calcium current in isolated rat ventricular myocytes. 50 and 100 µM MnCl_2_ significantly decreased the amplitude of L-type calcium current (Figures 4A,B). Thus, as previously described (see Introduction), manganese blocks L-type calcium channel. Given the major contribution of I_CaL_ in shaping cardiac electrical waveform, we next investigated the effect of MnCl_2_ on action potential.

### Manganese Decreases Action Potential Durations

**FIGURE 5:**
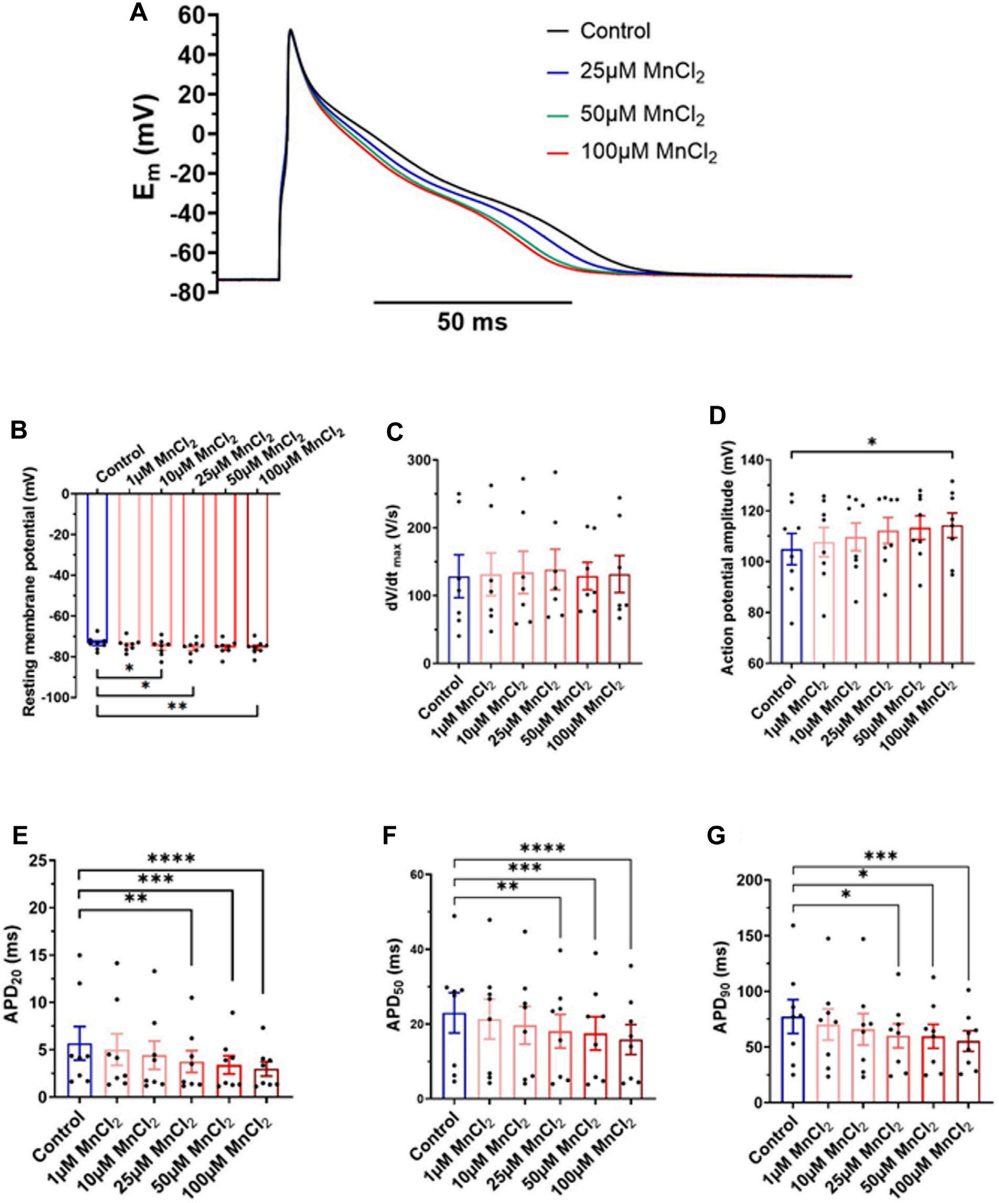
Effects of MnCl_2_ on action potentials of rat ventricular cardiomyocytes **(A)** Representative plots of action potentials in control condition and at 25, 50 and 100 µM MnCl_2_ **(B)** Resting membrane potential **(C)** dV/dt_max_ **(D)** amplitude of peak **(E)** APD_20_ **(F)** APD_50_ and **(G)** APD_90_ depending on different conditions (*N* = 4; *n* = 8). *p < 0.05, **p < 0.01, ***p < 0.001, ****p < 0.0001.

We recorded action potentials in rat ventricular myocytes under current clamp condition in the whole-cell configuration of the patch-clamp technique. Figure 5A shows shortening of the APD in response to ascending concentration of MnCl_2_. Mean data indicates a small but significant hyperpolarization (Figure 5B) of resting membrane potential at 10 µM (−2.7 ± 1.3%), 25 µM (−3.1 ± 1.3%) and 100 µM (−3.1 ± 1.2%). dV/dt_max_ was not impacted by MnCl_2_ (Figure 5C). AP amplitude was significantly increased at 100 µM MnCl_2_ only (Figure 5D), most probably due to the slight hyperpolarization. The major effect of MnCl_2_ was on APDs with a significant decrease from 25 μM of MnCl_2_ (Figures 5E–G). At 100 μM MnCl_2_, APD_20_, APD_50_ and APD_90_ were reduced by 36.4 ± 9.4%, 29.8 ± 5.1% and 23.4 ± 6.3%, respectively. Taken together physiological data at the whole heart and cellular level indicates that 25 μM MnCl_2_ does not induce cardiac toxicity. We next tested whether this concentration can be used in MRI measurements.

### Micromolar Manganese in Cardiac MRIs

**FIGURE 6:**
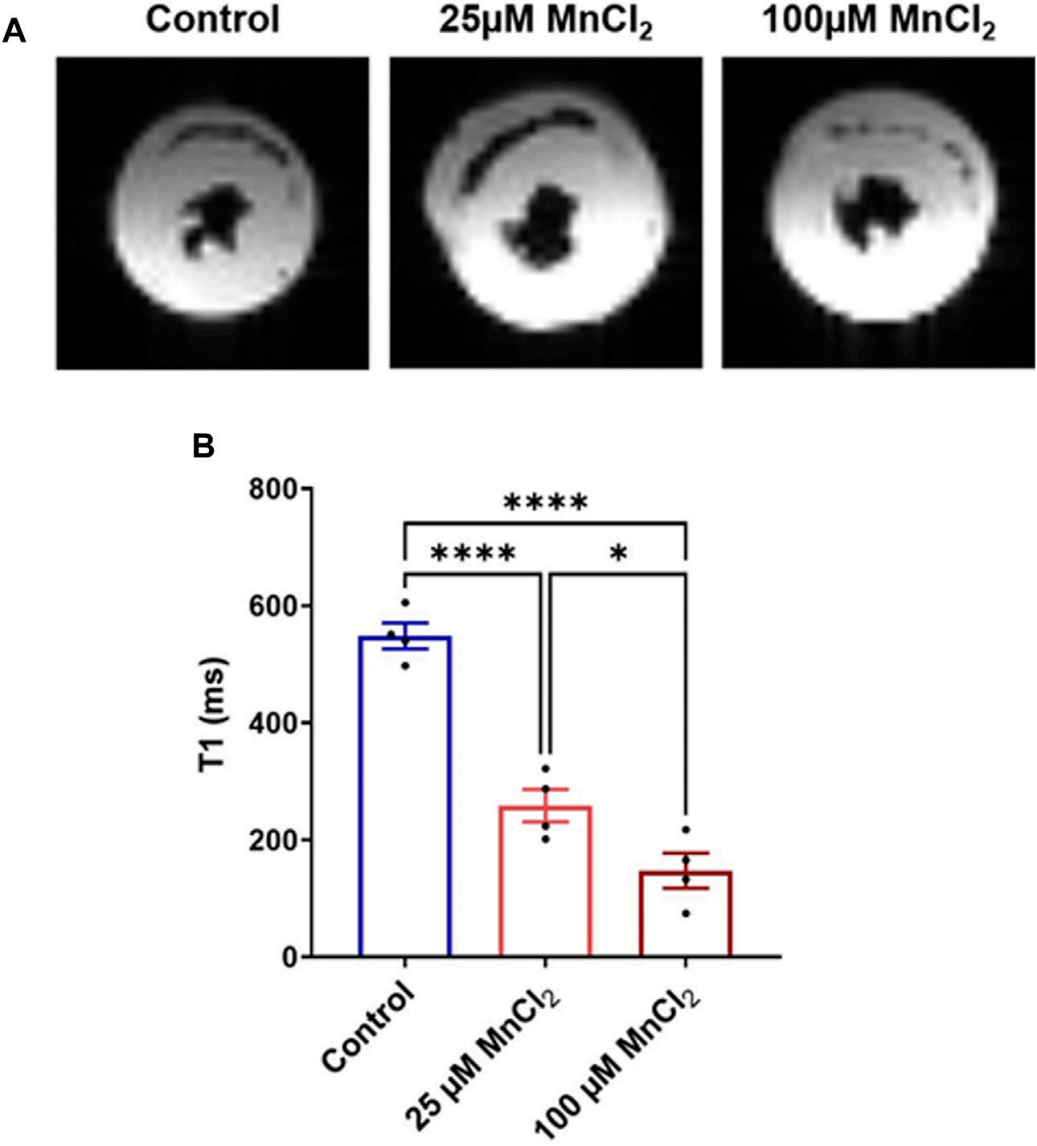
Effect of MnCl_2_ on T1 longitudinal relaxation time of myocardial tissue at 1.5T MRI. **(A)** Typical MR images of perfused rat hearts (mid-ventricular short-axis view) **(B)** T1 relaxation time of control condition (*N* = 4) compared to 25 µM (*N* = 4) and 100 µM (*N* = 4) of MnCl_2_. *p < 0.05, ****p < 0.0001.

To study the capacity of MnCl_2_ as a contrast agent in cardiac MRI at 1.5T, healthy rat hearts were perfused *ex vivo* in working mode with MnCl_2_ concentration of 25 μM (safe) and 100 μM (toxic). Figure 6A shows representative MRI images of rat hearts at the mid-ventricular level. A decrease in the longitudinal relaxation time T1 by 53 and 73% for rat hearts perfused with 25 and 100 µM of MnCl_2_ respectively was observed (Figure 6B).

## Discussion

Our study provides, for the first time, a comprehensive evaluation of manganese cardiac toxicity. Low micromolar concentration, up to 25 μM, have no impact at the whole heart level. At the cellular level, a small decrease in calcium transient amplitude and action potential duration is observed, mainly due to a block of L-type Ca channel. 25 µM MnCl_2_ decreases T1 in MRI experiments showing that this dose is sufficient for using manganese as a contrast agent.

### Experimental Approach

To evaluate the cardiac toxicity of manganese, we used two experimental approaches. First, the isolated heart model representing golden standard in basic cardiovascular research. Instead of the Langendorff heart model, we used the working model that is closer to the real physiological situation: the heart is perfused orthogradely, and the left ventricle performs the pressure-volume work. In addition to hemodynamic parameters, it allows detailed and simultaneous measurements of electrical and contractile function (Olejnickova et al., 2015). To get insight into the mechanisms, we used single cells. First, HL-1 cell line which has been used by many investigators to study many features of cardiac physiology (White et al., 2004). Importantly, HL-1 cells are spontaneously beating, which was crucial in our experimental settings to avoid using external electrical stimulation via platinum electrodes over a long time (30 min) which can be problematic and can lead to exhaustion of the myocytes due to prolonged stimulation. In HL-1 cells, spontaneous activity allows manganese to enter cells at each beat. Manganese is known to quench fluorescent signal (*e.g.,* Fura-2), therefore we used the newly developed calcium fluorescent dye, Cal-520 with little to no quench by manganese. In the same vein, to the best of our knowledge, there is no indication that manganese affects calcein fluorescence. If the cell is dying, calcein is rapidly extruded from cells. Our results show some variability (Figure 2B) most probably due to the change in ROI to avoid bleaching, but no decrease in the signal as it has been shown in control experiments using saponin to permeabilize cells (Bratosin et al., 2005). Finally, we used acutely isolated rat ventricular myocytes, which are more physiologically relevant both structurally and functionally to the living organism. This allows us to get biophysical insight with patch clamp recording of I_Ca_ using a physiological holding potential (Brette et al., 2006) and also physiological insight by recording action potential.

### Impacts of MnCl_2_ on Hemodynamic Function

Perfusion of hearts *ex vivo* has shown that the deleterious impact of MnCl_2_ on hemodynamic parameters was limited. A reduction in left ventricular contractility and relaxation was observed, only at 100 µM of manganese. We also observed that cardiac functional parameters, including cardiac contractility, were rapidly restored following perfusion of hearts *ex vivo* without MnCl_2_. Our results are in agreement with a previous study showing a negative inotropy recorded on adult rat myocardial preparations as soon as 300 µM MnCl_2_ was added (Naoki et al., 1992). Surprisingly, after an initial depression, manganese causes a late increase of contractile force in tissue preparations from adult rats or guinea pigs (Naoki et al., 1992; Tanaka et al., 2002). However, the mechanism of action associated with this late increase of contractile force is unclear (Vierling and Reiter, 1979; Dudek and Pytkowski, 1991). In our study, we do not observe this increase, most probably due to the use of lower MnCl_2_ concentrations. In addition, Brurok et al. (1997) observed an alteration of cardiac function above 100 µM manganese in rat hearts perfused in Langendorff mode, and the heart rate was decreased at concentrations above 1 mM. This is in agreement with the stability of heart rate that we obtained throughout the perfusion. This is consistent with an electrophysiological study in dogs showing that cumulative injections of MnCl_2_ (0.1–10 mg/kg, *i.e.,* ∼8.7–870 µM) does not affect heart rate (up to 1 mg/kg) (Charash et al., 1982). At doses higher than 1 mg/kg (*i.e.* ∼87 µM) changes in the heart rate and an increase QT, PR and QRS intervals was observed, but without any arrhythmia (Charash et al., 1982). Left ventricular contractility was partially restored when manganese was removed from the perfusion medium, suggesting that excitation-contraction coupling was also partially restored (bellow). Similar results were observed following a washout performed on hearts perfused in Langendorff with different concentrations of MnCl_2_ (1–3,000 µM) (Brurok et al., 1997).

### Impacts of MnCl_2_ on Excitation-Contraction Coupling

Alteration of the contractility observed at the whole heart level can be explained by modification of calcium homeostasis during excitation-contraction coupling at the cellular level. Our patch-clamp data indicates a blockade of L-type calcium channel (Ca_v_1.2) resulting in a decrease of I_Ca,L_. Such decreases will impact Calcium-Induced Calcium-Release mechanism and lead to a reduction of calcium transient (Bers, 2002), as observed in this work. This decrease in the systolic calcium concentration will in turn decrease the amplitude of sarcomere shortening, which explains the reduction in cardiac contractility observed with MnCl_2_ at the whole heart level. We do not observe an effect on the time to 50% relaxation of calcium transients suggesting that Na-Ca exchanger (NCX) and Calcium ATPase (SERCA) functions are not altered by manganese. Manganese enters the cytosol through L-type calcium channels and can be extruded by NCX as it was previously shown (Brommundt and Kavaler, 1987; Chen et al., 2012).

L-type calcium currents also play a major role in cardiac electrical activity (Bers, 2002). Our current-clamp experiments indicate a decrease in action potential duration at three APDs 20, 50 and 90. This is consistent with block of L-type calcium channels by manganese. This has been studied at the biophysical level as early as the 1970s in studies, and it appears that manganese has a specific affinity for the pore of calcium channels, partially blocking it (Payet, 1980). This block will lead to a decrease in APD as first shown by Ochi in 1976.

However, the effect on APD were small from a physiological perspective and we did not observe arrhythmic phenomena like early and late post-depolarization’s nor PVC.

### Use of MnCl_2_ as a Contrast Agent in Cardiac MRI

Our functional data indicates that MnCl_2_ up to 25 µM is safe to use as a contrast agent in cardiac MRI. Indeed, we showed that two concentrations of MnCl_2_ (25 µM safe, 100 µM toxic) reduced T1 on non-pathological cardiac tissue. A concentration of 25 µM is estimated at 229 μg/kg or 1.82 μmol/kg. Despite lacking comprehensive toxicological studies, manganese has started to be used for cardiac MRI in humans. Indeed, MnCl_2_ has been be used at 5 μmol/kg in healthy individuals resulting in an increase in T1 while ensuring short-term cardiac safety (Fernandes et al., 2011). In animal models, manganese uptake in myocardial tissue can be visualized with MEMRI and distinction between healthy and pathological areas (Hu et al., 2001; Krombach et al., 2004). Indeed, assessing myocardial viability can be assessed as only living cells will uptake manganese *via* L-type Ca^2+^ channel. MEMRI has been used at the pre-clinical level to allow quantification of infarcted area in animal models ranging from mouse to pig (Daire et al., 2008; Dash et al., 2011; Tada et al., 2019; Jasmin et al., 2021; Spath et al., 2021). Recently, two clinical trials were initiated to evaluate MEMRI in various types of cardiomyopathies. Today, only the results of studies with EVP1001-1 (unchelated manganese) are published, showing no adverse cardiac side effects observed during MEMRI (NCT01989195, NCT02933034). Results with Mn-DPDP (manganese chelate) are not yet published and/or are ongoing (NCT04623788, NCT01326715, NCT03607669).

The use of manganese in cardiac MRI appears to have great potential in imaging viable myocardium.

### Use of MEMRI in the Context of Heart Failure

MEMRI is also promising for diagnosing heart failure. Heart failure, a major health problem, is associated with a high risk of morbidity and mortality. Historically, heart failure has been defined by the inability of the myocardium to pump blood normally due to an impaired systolic contractile performance of the left ventricle (Mishra and Kass, 2021). Recently, a subtype of heart failure presenting clinical signs and symptoms of heart failure but with normal or nearly normal left ventricular ejection fraction has been identified. MEMRI could enable quantification of cardiac hypertrophy (by decreasing manganese uptake (Andrews et al., 2015)) and allows specific visualization of fibrotic areas (Su et al., 2014) with a T1 difference to healthy tissue in cardiac hypertrophic patients (Spath et al., 2020). In addition, MEMRI has the potential to monitor changes in intracellular Ca^2+^ homeostasis at the whole heart level. Thus, performing MRI with manganese-based contrast agents would optimize diagnosis and therapeutic decision-making in a patient by providing visualization of potentially arrhythmic areas.

## Data Availability

The raw data supporting the conclusion of this article will be made available by the authors, without undue reservation.

## Abbreviations

LGE, late gadolinium enhancement; MEMRI, manganese enhanced magnetic resonance imaging; MRI, magnetic resonance imaging.
